# Development of a universal psycho-educational intervention to prevent common postpartum mental disorders in primiparous women: a multiple method approach

**DOI:** 10.1186/1471-2458-10-499

**Published:** 2010-08-18

**Authors:** Heather J Rowe, Jane RW Fisher

**Affiliations:** 1 Centre for Women's Health Gender and Society, Melbourne School of Population Health, University of Melbourne, Victoria 3010 Australia

## Abstract

**Background:**

Prevention of postnatal mental disorders in women is an important component of comprehensive health service delivery because of the substantial potential benefits for population health. However, diverse approaches to prevention of postnatal depression have had limited success, possibly because anxiety and adjustment disorders are also problematic, mental health problems are multifactorially determined, and because relationships amongst psychosocial risk factors are complex and difficult to modify. The aim of this paper is to describe the development of a novel psycho-educational intervention to prevent postnatal mental disorders in mothers of firstborn infants.

**Methods:**

Data from a variety of sources were synthesised: a literature review summarised epidemiological evidence about neglected modifiable risk factors; clinical research evidence identified successful psychosocial treatments for postnatal mental health problems; consultations with clinicians, health professionals, policy makers and consumers informed the proposed program and psychological and health promotion theories underpinned the proposed mechanisms of effect. The intervention was pilot-tested with small groups of mothers and fathers and their first newborn infants.

**Results:**

*What Were We Thinking! *is a psycho-educational intervention, designed for universal implementation, that addresses heightened learning needs of parents of first newborns. It re-conceptualises mental health problems in mothers of infants as reflecting unmet needs for adaptations in the intimate partner relationship after the birth of a baby, and skills to promote settled infant behaviour. It addresses these two risk factors in half-day seminars, facilitated by trained maternal and child health nurses using non-psychiatric language, in groups of up to five couples and their four-week old infants in primary care. It is designed to promote confidence and reduce mental disorders by providing skills in sustainable sleep and settling strategies, and the re-negotiation of the unpaid household workload in non-confrontational ways. Materials include a Facilitators' Handbook, creatively designed worksheets for use in seminars, and a book for couples to take home for reference. A website provides an alternative means of access to the intervention.

**Conclusions:**

*What Were We Thinking! *is a postnatal mental health intervention which has the potential to contribute to psychologically-informed routine primary postnatal health care and prevent common mental disorders in women.

## Background

Mental disorders in women in the year after giving birth are diverse in nature and severity [1], have adverse consequences for individual women, their families and the health system [2], and continue to constitute a substantial public health burden in Australia [3] and elsewhere [4]. It is widely agreed that in addition to treatment for those affected, a comprehensive approach to mental health service delivery must include mental health promotion and the prevention of mental disorders [5].

Varied approaches to the prevention of postnatal mental health problems have been tested, including indicated interventions for women with current depressive symptoms, selective interventions for women identified by screening as at risk of developing depression, either by having current symptoms of mood disturbance or reporting exposure to known psychosocial risks, and universal interventions to be offered to all women to reduce population prevalence [6-8]. Universal strategies are preferred, because they can result in small changes in risk factors across a whole population, which have a greater public health benefit than treating individuals who are at high risk or already symptomatic [9]. Reviewers conclude that universal antenatal preventive interventions have been unsuccessful because of the low positive predictive values of screening measures, and because events after childbirth are more salient determinants [10].

Successful universal preventive interventions rely on clearly identified and modifiable risk factors, well theorised multifactorial models of causation, evidence based approaches to behaviour change, and non-stigmatising programs that can be delivered in primary care [6,11]. There are four well-established risk factors for depression after childbirth: personal psychiatric history, coincidental adverse life events, poor quality of relationship with the intimate partner and insufficient social support. Other risk factors for which there is less reliable evidence include poor physical health and adverse intrapartum experiences [12]. Despite the range of risk factors and theoretical mechanisms targeted in the trials of universal postpartum psychosocial interventions, prevention of maternal mental health problems remains elusive [8].

There are several potential explanations. First, postnatal mental health problems are multifactorially determined, and there are complex interactions among the biopsychosocial risk factors, and with individual variation in women's vulnerability to mental health problems because of psychiatric history or current circumstances [13] Second, some trials were initiated to provide evidence about feasible interventions that were poised for widespread implementation, rather than because the interventions were well theorised or expected to be effective [14]. Third, successful prevention in public health relies on a shared understanding and belief among stakeholders about the key risks and the possibilities that they present for prevention. Lumley (2005) suggested that the lack of involvement of mental health practitioners and researchers in prevention trials might have contributed to their failure [14]. Fourth, recognition of women's own conceptualisation of the causes of onset of depression and recovery is fundamental to the development of meaningful approaches to prevention, but was limited [14]. Finally, theoretical understanding of both treatment and prevention of mental disorders is growing in sophistication. Mrazek and Haggerty [6] conceptualise prevention and treatment of mental disorders as part of a continuum informed by similar multi-factorial causal models. Psychosocial treatments for which there is evidence of effectiveness in reducing morbidity, chronicity and disability, have promise for prevention, but have not been included in any of the universal interventions trialled to date.

The need for clinically effective, feasible and acceptable universal strategies to prevent common mental disorders in women after childbirth remains. These should address modifiable risk factors about which there is a shared stakeholder understanding and be based on clearly conceptualised multifactorial models of causation and behaviour change, non-stigmatising, and implementable in primary care. The aim of this paper is to describe the development of an innovative psycho-educational intervention to prevent common postnatal mental disorders including depression, anxiety and adjustment disorders in primiparous women.

## Methods

Multiple methods were required to investigate why existing prevention strategies, assessed in well-designed investigations, had not been successful, and to develop a novel and theoretically plausible intervention to be tested in a controlled trial.

### Review of the theoretical rationales of universal postnatal preventive interventions

The systematic reviews and the original reports of all trials of postnatal interventions implemented in unselected populations to prevent postnatal depression and anxiety, which have mental health as a primary outcome, were retrieved. More recent trials were identified through searches of PubMed, Medline, EmBase and Web of Science databases using the search terms listed in the systematic reviews: postpartum depression, prevention, postnatal depression, postnatal care, maternal mental health, postpartum mood disorder [8,10,13,15,16]. These were scrutinised by both authors to identify the theoretical rationales, conceptualizations of the condition to be prevented and the proposed psychological mechanisms whereby interventions would reduce prevalence of the disorder. Salient modifiable risk factors which had and had not been addressed were identified.

### Research evidence from Australian early parenting and community services

Australian parents and infants who are experiencing difficulties can receive treatment in residential early parenting services, which provide brief admissions to structured psycho-educational programs in both public and private health sectors [17]. Research evidence about the nature of presenting problems and follow up studies to assess effectiveness of treatments in early parenting services were identified and reviewed. This evidence enabled re-conceptualisation of modifiable risk factors, and identification of components of successful treatments for mental health problems which could be applied in primary prevention.

### Consultations with stakeholders

The intervention was designed to be provided as an integrated component of universal, standard primary maternal and child health care. In Victoria, the Australian state in which this intervention was developed, these services are provided by local government on behalf of the state government Department of Education and Early Childhood Development. We undertook purposive sampling to ensure representation from mothers and fathers of infants, consumers of postpartum mental health care, relevant professional primary health care and specialist disciplines, including general practice, lactation consultant, maternal and child health nursing, paediatrics, psychiatry, psychology, early parenting services staff and local government service coordinators. Stakeholders comprised an expert reference group which met on three occasions during which discussion was facilitated by the authors. Detailed minutes of the discussion were recorded by a research assistant and later transcribed. Data were also collected in individual semi-structured interviews, conducted by at least one of the authors, with practitioners who were not present at reference group meetings. Detailed field notes were recorded. Minutes and field notes were scrutinised independently by each of the authors. All themes were identified and coded. Any discrepancies were resolved in discussion. These consultations were deemed a health service quality assurance activity for which ethics approval was not required.

### Psychological and health promotion theories

We identified and articulated theories of causation of anxiety and depression which accorded with women's understandings of their predicaments, offered opportunities for intervention, and provided plausible mechanisms for effectiveness. Relevant theory was applied in the development of the intervention.

### The *What Were We Thinking! *intervention

Intervention materials were developed, program theory, content and methods of delivery were documented, qualified maternal and child health nurses were trained as facilitators, and the intervention was pilot tested with two groups of parents. Approval to conduct the pilot study was given by Victorian Department of Human Services Human Research Ethics Committee and Masada Private Hospital.

## Results

### Theoretical rationales of universal postnatal prevention interventions

The 7 published trials of universal postnatal interventions to prevent postnatal mental disorders in women, which have mental health as a primary outcome, and 3 relevant systematic reviews were retrieved. The mental health outcomes assessed in each trial, and the supporting psychological mechanisms and risk factors that were addressed in each intervention were identified.

Postnatal preventive interventions designed for universal application in unselected populations have included stress debriefing after childbirth [18,19]; an early postnatal check-up [20]; assistance from community-based postnatal support workers [21] and provision of a self-help manual with an invitation to attend a support group [22]. Of these, only the midwife-listening visit trial [19] reported a beneficial effect. However, there was apparent selection bias in that 60% of participants were single women, and the very high rates of self-reported depression and anxiety in the control arm (50% classified as having clinically significant symptoms) led reviewers to conclude that it is a 'true outlier' [8]. The other two trials assessed comprehensive community-based interventions and only the resource-intensive, protocol-based midwifery-led care focussed on individual women's physical and psychological needs, conducted in the UK National Health Service where women are already likely to have existing relationships with primary care physicians, resulted in a reduction in symptoms of depression [23]. The other trial of an Australian program of resources, information and support for mothers (PRISM), enhanced with general practitioner training and focused community development [24] did not. All studies were adequately powered, analyzed by intention to treat, had properly concealed random allocation to trial arms and blinded assessment of outcomes. There were some methodological limitations: attrition greater than 20% at final assessment [20-23] and in one trial there was poor compliance with the intervention, raising the possibility that women most in need may not have had the opportunity to benefit [22]. Dennis [25] concludes that these studies are generally of good methodological quality. The findings raise questions about why most of the interventions were not effective. A number of explanations emerge.

#### Conceptualization and measurement of mental health outcomes

All of the interventions were intended to reduce depression and four [20-23] also assessed general psychological distress and role functioning using the SF-36 [26]. Few studies addressed the possibility that 'postnatal depression' might not be a distinct construct and that other mental health conditions might be salient outcomes. One assessed anxiety as well as depression [19], using the Hospital Anxiety and Depression Scale and reported scores on both subscales. Priest et al [18] assessed both post-traumatic stress disorder and depression and were the only group to use a diagnostic outcome assessment in a two-stage process involving self-report symptom measures and the clinically-administered Schedule for Affective Disorders and Schizophrenia (SADS) [27] with sub-groups. Most used the self-report Edinburgh Postnatal Depression Scale (EPDS) [28] to assess depressive symptoms and compared both mean scores and proportions scoring in the clinical range.

There is increasing evidence that postnatal anxiety disorders are at least as common, but less well recognised than depression [29-31]. The EPDS is now known to detect anxiety disorders as well as depression, but its capacity to distinguish the conditions from each other is not established [29,32-34]. Brockington [1] in a review of postnatal psychiatric disorders concludes that women identified through screening as depressed actually have heterogeneous conditions including posttraumatic stress disorder, panic, phobic, obsessional and generalised anxiety disorders, adjustment reactions and depression. These are situation-focused, disabling and often reflect adversity [35]. Brockington [1] argues that 'postnatal depression' therefore has value as a lay term, but is imprecise as a clinical or a research construct. Evidence that anxiety is a salient outcome is provided by a randomised controlled trial of an enhanced childbirth education program [36] which found improvements in anxiety, assessed using the Spielberger Trait State Anxiety Inventory [37], at 6 weeks and 6 months postpartum in women in the intervention group compared with a standard childbirth education group. It is possible that mental health benefits of existing interventions were missed because salient outcomes were not assessed.

#### De novo or recurrent mental disorders

Another possible explanation for the failure of most universal interventions to exert an effect on postnatal mental health is variation in vulnerability to mental disorder in populations of women. In their comprehensive prospective study of 646 primiparous married women recruited systematically from a general maternity service, Cooper and Murray [38], found that 61 had experienced major or minor depression in the first three months postpartum. In 40 (66%) of these women it was a first onset or *de novo *condition, and in 21 (34%) it constituted a recurrence of previous "affective disturbance". They found that depression was of shorter duration in the *de novo *than the recurrent group and concluded that adaptive capacities might also differ between these groups. None of the trials of universal interventions investigated differences in effectiveness of the intervention in women with and without a psychiatric history [8].

#### Psychological mechanisms for the universal interventions

Varied implied or explicit psychological mechanisms, reflecting different theoretical models of causation, informed the existing prevention interventions. One group of studies addressed social support. These postulated that unmet maternal emotional needs would be fulfilled by well-informed empathic professional support from a midwife or befriending by a trained community postnatal support worker [21,23,24]; that the provision of pertinent information to increase knowledge about infant behaviour and normalise maternal needs for support, and encouragement to participate in facilitated mothers' groups would reduce social isolation [22]; and two postulated that cognitive and emotional integration of adverse psychological sequelae of intrapartum experiences would be assisted by standardised listening interviews with a health professional [18,19].

Gunn et al [20] hypothesised that symptom duration and distress would be reduced by early identification of and response to maternal health needs by a general practitioner. The large scale primary care and community-based interventions also theorised that sensitised and specifically-skilled primary health care professionals would detect women's needs earlier and respond more effectively than those who had not been trained [23,24]. In addition, the comprehensive program of resources, information and support for mothers (PRISM) intervention was based on social ecological theory, including components positioned at the levels of the individual and the environment. The investigators proposed that maternal mental health is optimised by accessible and high quality local environments and by improved social recognition of mothers, including advocacy for their needs [39]. Insufficient social support is an established risk factor for postnatal mental health problems [12], but the results of universal preventive trials indicate that addressing this with enhanced professional support or improvements in women's local environments do not improve maternal mental health.

#### Women's understanding of mental health problems

The effectiveness of existing interventions might have been limited because women's own detailed understandings of the causes of their mental health problems or their recovery were not included in the theoretical rationales. In a landmark study which followed 45 women up 12-18 months after they had participated in a population-based postnatal health survey and scored more than 12 on the EPDS, Small and colleagues [40] found that women most commonly attributed their distress to feeling unsupported, being in poor physical health and tiredness. Women who had recovered since the first survey attributed this to the baby getting older, having more support from their partners, returning to paid work and not feeling so tired. Many participants regarded their "depression" as not to do with their babies, but because of their circumstances, and rejected psychiatric labelling implicit in the term "postnatal depression".

#### Stigma and participation

There is little information reported in the available trials regarding what participants were told about the interventions in which they participated. While public education programs in Australia have reduced the stigma associated with psychiatric conditions [41], most people do not regard themselves as at risk of mental disorder and the use of psychiatric labels and constructs can be a barrier to participation in primary care mental health programs [11]. Mrazek [6] argues that there is a need for interventions which use health promotion language and concepts rather than illness labeling. Such programs have the potential to engage vulnerable groups in mainstream healthcare where their problems may be readily identified and appropriate referrals to specialist services made.

#### Modifiable risk factors that have been addressed in universal interventions

The goal of preventive interventions is to address modifiable risk and protective factors [6]. Of the four well-established risk factors for postnatal mental health problems in women, psychiatric history and coincidental adverse life events are the least readily modified in the postpartum period. Most of the universal interventions addressed low social support, which is one of the more readily modified risks, and three addressed risks for which there is lower level evidence: poor physical health [20] and adverse intrapartum experiences [18,19]. Each of these attempted to modify factors in individual women, and only the PRISM study sought to reduce depression by modifying the social environment in which mothers lived [24].

#### Modifiable risk factors that have not yet been addressed in universal interventions

Chen et al [42] provide evidence that depression in mothers is attributable more to social than to psychological or biological factors. However, current conceptualisations locate postpartum mental health problems within individual women and position fathers and infants as being affected by rather than potential contributors to these difficulties [43]. At this life phase women have increased dependence on intimate relationships, and reduced interactions with workplaces and the broader community. We postulate that depression and anxiety in mothers of newborns can be conceptualised as reflecting poorly functioning intimate relationships, which are potentially modifiable.

##### Intimate partner relationship

There is consistent evidence that the quality of relationship with the intimate partner is associated with postnatal mental health in women. It has been found to act both protectively and to increase risk. Women who experience their partners as welcoming the pregnancy, and providing empathic support and encouragement, have better mood [44-46]. In contrast, women who feel unable to confide in their partners, or are experiencing conflict, poor communication or dissatisfaction in the relationship have worse mood [44,47-55]. There is little consideration in this literature of the contribution of critical coercion, intimidation and other forms of emotional and physical abuse to postnatal mental health problems, but in general, gender-based violence is a central determinant of depression and anxiety in women [56].

Although the evidence is consistent, few investigators have operationalised how non-specific difficulties in the intimate partner relationship are enacted in day-to-day behaviors. Only two universal interventions for the prevention of postnatal mental health problems, both brief and offered during pregnancy, included partners. Fifty years ago Gordon et al [57] included men in two additional childbirth education classes, not only modelling that the work of parenting is a shared obligation, but also providing guidance to sensitise men to the demands of this life change for women. Significantly fewer 'emotional upsets' were found in women in the intervention than the standard care group six months postpartum. More recently Midmer [36] conducted a randomised controlled trial of two additional 3-hour classes, conducted mid-pregnancy, or standard childbirth education program for women and men. The classes focussed on increasing couples' appreciation of new mothers' common feelings of isolation, ambivalence, conflict, resentment and guilt, and gaining skills for managing pressures from extended family, a fretful baby, and the redistribution of household chores. Problem solving and communication techniques were practised in role play activities. When followed-up 6 weeks postpartum, women and men in the intervention group had significantly lower anxiety than those who received standard childbirth preparation and the effect was sustained at 6 months postpartum. Although there are methodological flaws in each study [8], including failure to adjust for cluster randomisation [36,57], use of non-standardised outcome assessments [57], attrition and response bias [36], the results provide some evidence that the inclusion of partners might be a promising approach to prevention

##### Persistent infant crying and unsettled infant behaviour

To date, most investigations in this field have presumed that infants' behaviour reflects parenting factors [2], in particular that prolonged infant crying is a consequence of maternal depression [58]. Few have acknowledged that the relationship might be reciprocal and that infant behaviour might exert an adverse effect on a mother's confidence and affect. Infant behaviour, especially prolonged inconsolable crying, frequent night-time waking, short daytime sleeps and feeding difficulties are very common reasons for mothers of infants to seek help [59,60].

Although crying is normal in infants, there is wide individual variation in the amount and intensity of infant crying and fussing in the first year of life [61]. Problematic or excessive crying is conceptualised as the upper end of a continuum of infant crying that is not tolerable to caretakers [62] and occurs in 10 per cent to 30 per cent of infants in the first three months of life [63,64]. Excessive, prolonged, inconsolable infant crying is usually difficult to explain and in systematic investigations has been found not to reflect pathological care taking or an abnormal familial environment [65]. Mothers of infants who cry excessively report significantly more parenting stress and less sense of competence and efficacy than other mothers, and do not experience their infants as a source of positive reinforcement [63]. Excessive, inconsolable infant crying and resistance to comforting is associated with earlier cessation of breastfeeding, frequent changes of infant formula, maternal irritability, poorer mother-infant relationship and heightened risk of infant abuse [62].

Infant sleeping behaviours can also be problematic. Approximately 46 per cent of an Australian community sample of parents described a sleep problem in their 6-12 month old infant including co-sleeping, being dependent on suckling to go to sleep, prolonged time to settle, frequent overnight waking, prolonged crying and short daytime sleeps. Infant sleep problems were associated with maternal symptoms of depression when other risk factors were controlled for [66]. The causes of infant sleep difficulties are not clear, but frequent waking and resistance to settling often co-occur [67].

Both infant and parent factors appear to be involved in the development and maintenance of dysregulated sleep. There is wide variation in sleep-wake patterns and self-soothing capacity [68] and consistent evidence that infants with the temperamental characteristics of a low threshold to arousal, intense reactions to unfamiliar stimuli and high levels of motor activity are more likely to have poor quality and dysregulated sleep [59]. Infant sleep is also influenced by parental caretaking. Infants placed awake in bed are more likely to learn to self-soothe and to have longer continuous sleep periods [68], and babies who have more than 11 feeds in 24 hours at one week of age are less likely to sleep through the night at 12 weeks of age than infants who had fewer feeds [69] Burnham et al [68] propose a transactional model in which both parent and infant factors interact in a dynamic process, in which behaviours are moderated through interactions with caregivers. None of the universal interventions addressed infant behaviours or included the infant.

##### Occupational fatigue and excessive maternal workload

Severe occupational fatigue is associated with prolonged or irregular working hours, particularly with early starting times and overnight work, which disrupts the circadian rhythm. It is especially problematic in highly mentally and emotionally demanding occupations in which there are inadequate rest breaks [70]. Occupational fatigue has adverse effects on emotional, cognitive and physical domains and severe fatigue leads to increased irritability, agitation, reduced empathy and sociability, low mood, and a general loss of insight and self awareness [70].

Late pregnancy and the first postpartum year are characterised by substantial disruptions to maternal sleep patterns necessitated by attention to infant overnight feeding and settling needs [71]. This is associated with impaired mood and cognitive function [72], which is more severe in primiparous than multiparous women [73], and may be implicated in the onset of postpartum depression [74]. Dennis and Ross [75] found in a longitudinal study of 505 women who scored less than 13 on the EPDS [28] at 4 weeks postpartum, that self-reported fatigue, having fewer than 6 hours sleep in a 24 hour period, and frequent infant crying were significant predictors of higher EPDS scores at 8 weeks. The authors suggested that interventions to prevent postpartum depression should include measures to reduce sleep deprivation in the early postpartum period. However, occupational fatigue has rarely been considered as a mediating factor for poor maternal psychological functioning after childbirth and was not addressed directly in any of the universal interventions.

Smith and Ellwood [76] sought to quantify the unpaid 'caring workload' in the Australian National University Time Use Survey of New Mothers which includes a non-clinical sample of 162 mothers of infants recruited from the community. At infant age three months there were on average 49 breastfeeds a week which lasted a mean of 20 minutes but up to 33 minutes. On average there were 70 other occasions a week when mothers were carrying, holding, or soothing their infants for around 13-18 minutes each time. Smith and Ellwood [76] conclude that active and passive infant care occupy about 23 hours of each day in the first year after birth.

Prevailing gender stereotypes about work mean that mothering an infant is not dignified with the language of work, the domestic setting is not conceptualised or named as a workplace and therefore does not benefit from occupational health and safety assessments.

In summary, there are several possible explanations for the overall lack of success of interventions for the prevention of mental health problems in women with infants, including neglect of potentially-modifiable risk factors. We argue that preventive initiatives should focus on modifiable risk factors, including intimate relationships, reduce occupational fatigue, recognise that psychiatric history influences individual vulnerability to mental health problems and address anxiety as well as depression.

### Research evidence from Australian early parenting and community services

Studies investigating the presenting needs of women admitted with their infants to residential early parenting services include that anxiety symptoms are more prominent than depressive symptoms [34] and that many women adhere to gender stereotypes about the division of domestic labour, lack the language and interpersonal skills to negotiate fairer arrangements, and feel criticised by their partners and mothers-in-law about the quality of their infant care and household management [77]. Occupational fatigue associated with interrupted and insufficient sleep is pervasive and disabling. Infant crying is a powerful anxiety-arousing stimulus and many women respond immediately with emotional and avoidant strategies [78]. In these services, solution-focused responses to infant crying are taught readily and rapidly through supported exposure to infant crying, education to promote situationally-informed interpretations of the baby's needs and training in settling skills [17].

#### Mental health problems in mothers at admission

Eight surveys of consecutive cohorts of women admitted to residential early parenting services in Australia have been reported [34,77,79-84]. All have found high prevalence of depressive symptoms and in three studies, 25% to 31% of participants met diagnostic criteria for depression or anxiety [82,83,34]. Fisher et al [77] found that almost all (91%) had clinically significant fatigue. Oddy et al [85] surveyed women 12 months after discharge from one of the services, half of whom attributed their distress during admission to difficult infant temperament and behaviour, fatigue, and inadequate practical support.

#### Infant characteristics on admission

Problematic infant behaviour, including frequent overnight waking and long periods of crying and fussing, is almost universal in infants admitted to early parenting services [80,81,84,86-88]. Feeding difficulties, prior diagnoses of gastro-oesophageal reflux [80,89] and significantly higher ratings of difficult infant temperament than population norms are also common [88].

#### Correlates of maternal mood

In this group of studies, poorer maternal mood was associated with worse infant sleeping difficulties [81], higher maternal anxiety symptom scores with difficult infant temperament [82], and more severe symptoms of depression and anxiety with prolonged infant crying and partners in whom women felt unable to confide [84], or who displayed criticism and a lack of empathy [77]. Low paternal participation in infant care and household work was associated with prolonged hours in de-regulated workplaces and with the pursuit of independent leisure activities that did not include the woman and her baby. All participants who reported experiencing physical violence in the previous year had EPDS scores in the clinical range and were the group least likely to recover in the short term [89].

#### Evidence of women's mental health after admission

Five prospective cohort studies assessing the effects of brief admissions to Australian residential early parenting centres on maternal mood, sense of maternal efficacy and psychological functioning have been published. All have shown significant improvements on all dimensions at follow up one to six months post discharge, including reductions in depression symptom scores [80,84,89-91], anxiety symptom scores [84,88,89,91] and proportions with clinical exhaustion [84,88,89]. Significant improvements were also observed in self-rated confidence in infant care [84,89,90] and women's perceptions of the experience of motherhood and emotional attachment to her infant [91].

#### Evidence of infant behaviour after admission

All these brief structured psycho-educational programs address unsettled infant behaviour. This involves teaching sleep behaviour management strategies to reduce unsustainable sleep associations, provide low stimulus comfort while the infant is learning to settle to sleep and establish a routine of regular day time sleeps in addition to reducing night time feeds at an appropriate age. The strategies are taught via individualised programs, hands on practice and active professional support, and provision of written information for ongoing reference. Six studies investigated changes in infant behaviour [80,84,87,88,91,92]. In all, mothers reported significant improvements in overnight waking, total crying and fussing, infant distress when put to bed, and dependence on sleep associations, sustained at three or six month follow up.

#### Management of unsettled infant behaviour in community services

There have been a number of randomised controlled trials of interventions to manage infant sleep in community settings. Of the trials that were attempting to treat variously defined clinical populations in which infant sleep was problematic, two used increased support, but no specific behavioural training and found no benefits to this approach [62,93]. Five others used training in a range of infant behaviour management strategies as a trial arm, comparing it to support, information leaflets, co-sleeping with a parent for a week or pairing it with placebo medication [62,94-96] and one [97] had a control group randomised to receive only routine care. All utilised individual consultations either in a clinic or by telephone, with a trained volunteer or a specialist clinician. Although not directly comparable, because different outcome measures were used, all reported that training parents in behaviour management strategies to assist infant sleep disturbance was effective in reducing either infant crying, or frequency of overnight waking, or resistance to settling, or requiring parental presence to fall asleep, or some combination of these. Furthermore, managing infant sleep problems has been shown to reduce maternal symptoms of anxiety [94] and depression [96,97].

Overall the results of these trials are consistent with the findings of the prospective studies, that neither non-specific support or information pamphlets appear to be effective in assisting unsettled infant behaviour or dysregulated sleep. However, training in infant behaviour management strategies is effective and leads to improvements in infant manageability and family emotional climate, in particular maternal mood, which are sustained at least in the short-term. These successful treatments provide promising avenues for the development of preventive interventions.

### Consultations with stakeholders

Successful interventions rely on a shared understanding of risk and protective factors amongst relevant stakeholders [14]. Parents of newborns, including past patients at residential early parenting programs and from the community, indicated that while there was ample, even excessive information provided about breastfeeding, there were high unmet needs for information about and skills to manage infant crying and sleep and settling in the early postpartum period. Men reported consistently that once the baby was born they felt excluded from standard maternal and child health care, some described discomfort attending primary health clinics and that their infant care skills were not fostered and remained under-developed.

Health care practitioners including paediatricians, maternal and child health nurses, general practitioners and lactation consultants described unsettled infant behaviour and maternal anxiety and fatigue as common presenting problems and endorsed a specific psycho-educational approach to early intervention. They affirmed the clinical salience of a gender-informed explanation of maternal mental health problems, and welcomed the opportunity to contribute to the development of a universal intervention that involves fathers.

In one local government area a maternal and child health care initiative involving a half day structured seminar on infant settling using didactic presentations and demonstrations of the skills had been established for several years. It was specifically designed to include both parents and offered at infant age four months in group format. Parents were informed of the program at the standard first home visit by the maternal and child health nurse. Participants evaluated the program as highly relevant and acceptable and the local government department regarded it as clinically and economically effective. Overall, the findings established a shared understanding of the theoretical rationale and feasibility of implementing the intervention in primary maternal and child health care.

### Psychological and health promotion theories to inform a psycho-educational approach

Psychological and educational theories were synthesised to inform the approach.

#### Causal framework for depression

Brown and Harris's [98] social theory proposes that depression in women arises from experiences of entrapment and humiliation, which we argue are salient to the circumstance of mothering a newborn. The work of infant care is intrinsically confining. If the baby is responsive and rewards the mother by quieting to her soothing, smiling, interacting, suckling easily, and developing along at least an average trajectory, the baby provides gratification. In contrast, an infant who resists soothing, cries inconsolably, or is difficult to breastfeed can be experienced as critical and unappreciative. The work of mothering an infant and managing a household in which an infant lives is repetitive, isolated, never complete, and can be ungratifying. A mother of a newborn depends on her partner for recognition and affirmation of her endeavours and is especially vulnerable to his criticism, which can be humiliating.

Disenfranchised or unrecognised losses can lead to grief and depression [99]. When a woman has a first baby she experiences overnight the losses, at least temporarily, of occupational identity, capacity to generate an income, social and leisure activities, bodily integrity, autonomy, and liberty. There is little public recognition of these losses, no ritual to acknowledge them, and often an insensitive and envious response which suggests that mothering a baby is easier and more gratifying than paid employment. Stereotypically, there is a marked gender difference in disenfranchised losses arising from the birth of an infant. Men can lose very little and might, in fact, participate less in domestic life after the birth of a baby than before. This brings about a gendered divide in daily experience that was unanticipated and proves difficult, at least initially, for most couples to recognise and negotiate fairly.

#### Causal framework for anxiety

Cognitive theories of anxiety are that it arises in situations in which a person feels helpless and unable to exert an effective response [100]. Caring well for an infant is sophisticated and technically skilled, requiring maturities of emotional responsiveness and sensitivity, in addition to knowledge of a newborn's developmental capacities. Few women have these competencies when they first become mothers and all mothers have to manage anxious arousal as part of becoming skilled at infant care and forming a confident maternal identity.

Infant crying is a powerful stimulus for anxious arousal. The most common response in inexperienced mothers is to respond emotionally and use multiple techniques to reduce the crying. When these are not effective, parents can use an increasing range of unsustainable strategies in order to avoid the crying and anxiety can intensify. Unsettled infant behaviour leads to repeated help-seeking, including from informal sources. Fashions of infant care change with knowledge, and practices from previous generations are not always congruent with current best practice. Reponses often constitute opinion rather than evidence and can be both ineffective and tinged with criticism, thereby contributing to increased anxiety. Women have high learning needs, to which a common response is just to trust their intuition, but the nature and formation of maternal intuition is generally unquestioned by health care providers or consumers. This might be sufficient for a well-resourced mother caring for a temperamentally easy baby, but if the mother is poorly supported and the baby is difficult to feed, settle or soothe, a mother can feel ineffective and powerless and a cycle of amplifying anxiety can develop.

#### Occupational fatigue as a mediator of depression and anxiety

Occupational fatigue reduces resilience, functional efficiency, cognitive clarity and capacities for emotional regulation and effective problem solving [70]. Occupational fatigue in mothers of newborns is associated with frequently interrupted and overall insufficient sleep, an excessive workload and minimal leisure time [77]. It is amplified by conformity to stereotypes about what constitutes work. The prevailing stereotype, reflected in language used in social settings and healthcare consultations is that paid employment is defined as work and mothering responsibilities as 'not work'. Pregnant woman are asked when they are 'giving up work' and mothers of newborns when they are 'going back to work' carrying the clear stereotyped message that infant care is not work. Many women assume this increased workload singlehanded and seek to spare their partners who 'are working'. They lack the concepts, language and interpersonal skills to name and then re-negotiate the workload and to acknowledge their fatigue as legitimate, disabling and requiring a shared response.

We propose that adaptation to motherhood is compromised and vulnerability to anxiety and depression increased by severe occupational fatigue and that the effect of this mediator has to be taken into account in preventive interventions. Postnatal affective disturbance reflects functioning in intimate relationships and that women desire care from and gratification within these relationships rather than increased care from health professionals.

#### Health promotion and education theory

Theories of group behaviour, adult learning and social cognition were identified as salient to a psycho-educational approach to prevention of mental health problems. Psycho-education seeks to improve knowledge about relevant risk and protective factors for mental health problems, builds on participants' strengths and resources, and promotes specific skill development.

Adult learning theory proposes that adults are motivated to learn when their need to know is aroused; they are self-directing and autonomous, and motivated as much by personal reward as by the intrinsic value of new knowledge; their orientation to learning is problem-centred and contextual; their prior experience is an important resource and they have well-differentiated learning styles which should be accommodated by multiple learning strategies [101]. Opportunities to "learn through doing" in active practice are powerful processes to promote skill acquisition.

Theories of small group instruction postulate that groups of adults at the same life phase can form socially cohesive environments which are conducive to learning by promoting exchange of knowledge, sharing of emotions and constructive critique of attitudes and norms [102].

Social cognitive theory states that awareness of risk and protective factors is a pre-condition of changed behaviour [103]. Our psycho-educational approach to mental health promotion for first time parents relies on communicating comprehensibly about novel modifiable risk factors, and supporting the development of a set of well-operationalised skills to realise change in behaviour [103].

Together these theories underpin our conceptualisation of maternal affective state as being dynamic and governed predominantly by interactions within intimate relationships mediated by occupational fatigue, and that new parents' learning needs about these factors will be addressed best using a psycho-educational intervention.

### The *What Were We Thinking! *intervention

*What Were We Thinking! (WWWT) *is a novel, highly structured, psycho-educational program for mothers, fathers and their first newborn intended to promote confident parental caretaking, optimise infant manageability and family functioning, and reduce common postnatal mental disorders in women.

#### Theoretical rationale

The theoretical principles of the *WWWT *program are that postpartum anxiety is as important as depression and requires explicit attention, but as depression and anxiety are not easily distinguished, they are addressed most effectively together; that partner and infant behaviours can be modified to decrease interactions that contribute to maternal depression and anxiety, and increase those that promote maternal confidence and competence; that improvements in ongoing day-to-day interactions are of fundamental importance to mental health promotion; that this knowledge needs to be made available at a critical developmental stage and in a readily comprehensible form, and that the language of the intervention needs to challenge gender stereotypes and honour the work of mothering newborns, to emphasise the universal nature of these tasks, and to describe this in non-psychiatric language as addressing "parenting confidence".

*WWWT *accords with recommended best practice for preventive interventions because it is designed to be integrated into existing community health care, [8,104] and offers more than just supportive listening or social support [105]. The program aims to reduce risk and strengthen protective behaviours through providing couples with specific understanding and skills about infant caretaking and managing the alterations to the intimate partner relationship after the birth of a first baby. It addresses theoretically plausible psychological mechanisms of causation of postnatal depression and anxiety by aiming to minimise experiences of humiliation through increasing father's understanding and empathy; counter experiences of entrapment by promoting infant care as a shared endeavour in which parents with comparable competence can permit each other independent or shared leisure and promote cognitive-rather than emotion-focussed responses to infant crying by building skills to respond in non-avoidant ways. Together these strategies are expected to promote gratifying and rewarding intimate interactions rather than frustrating and diminishing ones, and thereby lead to increased parental confidence, more settled infant behaviour and reduced depression and anxiety.

#### Primary health care setting

In Australia, antenatal education for women and their partners offered in hospital settings is well established, non-stigmatised and participation by expectant parents is high. Existing curricula provide preparation for labour and birth, and there is increasing recognition that these programs do not adequately prepare parents for the responsibilities of infant care or relationship adjustments necessary in the early postpartum period [106]. Many prospective first-time parents report worry about their parenting competence, the effect of the baby on their relationship and fears about the mother's potential isolation in the home [107,108]. Understandably however, their attention during antenatal classes is predominantly focussed on the impending birth, and additional classes in the postnatal period are necessary to achieve improvements in specific parenting-related outcomes [106,109]. Parenting-related material has been offered in a "postnatal reunion" class offered in hospital as an extension to the standard antenatal education program [106], but new parents prefer to attend groups established in their local communities because of the opportunities for the formation of lasting social networks amongst neighbours [110].

In the Australian state of Victoria, First Time Parent Groups are offered as part of standard Maternal and Child Health Services [111]. Participation varies by location from about one third to over 80% of first time mothers, but few men attend [112]. The primary aim of these groups is to foster social support, but they provide an established infrastructure for the location of a group-based program to address specific known risk factors for postpartum anxiety and depression in a format that is non-stigmatising, that is interactive and informed by adult learning principles, is purposely designed to include men, and that also meets new parents' learning needs and fosters social support amongst them.

#### Psycho-educational approach

*WWWT *is a highly interactive program for mothers, fathers and their infants, which aims to provide salient knowledge and opportunities to learn new skills. These are difficult to acquire through self-learning at this life stage because of fatigue and because it is hard for most people to distinguish between the written resources that are evidence-based and those that constitute personal experience or opinion. *WWWT *includes group discussion, focussed tasks to be undertaken individually and then discussed as a couple, practice in problem solving and negotiation, hands-on supported practice in infant settling, short talks and practical demonstrations. Anxiety is contained by a supportive, non-judgemental and knowledgeable facilitator. Very careful language use is prescribed so that gender stereotypes are challenged, fathering and mothering are positioned as different but of equal importance, and emotions are named and normalised without the use of psychiatric labelling.

#### Specific content and materials

The *WWWT *program has 13 sections, grouped into two components: "About Babies" and "About Mothers and Fathers". The program contains information about infant temperament, crying and sleeping, and sustainable strategies for promoting settled infant behaviour, new language and concepts about the losses and gains of first-time parenthood and re-negotiating the increased unpaid workload.

A creative producer, experienced in designing materials to communicate complex information through images and simple language was contracted to assist with development of materials. This was an iterative process including consultations with the key stakeholders. The materials are attractive, inclusive, illustrative, easily read and understood and contain clear key messages. A folder containing a booklet about program content and interactive worksheets for each section are used during the program and taken home by couples for later reference. The *WWWT *program theory, learning outcomes, group strategies and interactive worksheets for each section are contained in a comprehensive Facilitators' Handbook.

#### Training and supervision of facilitators

The program is designed to be conducted by qualified Maternal and Child Health Nurses, experienced in group facilitation. Specific learning needs of facilitators for information about the underlying theory, the use of gender-sensitive language and supporting parents in practising infant settling are addressed in a half-day training session.

#### Program delivery

The program is designed for delivery in one half day session in local, readily accessible maternal and child health centres, with groups of up to five couples and their four to six week old babies, on Saturdays in order to maximise participation of fathers.

#### Pilot testing and modification of the intervention and materials

The intervention was pilot tested with eleven couples and their infants in two separate groups. The mean age of participants was 31 years, and they were English speaking, predominantly well-educated and in professional occupations. The anonymous participant satisfaction surveys affirmed the salience, timing and value of the approach for first time parents. Participants' suggestions for increased clarity and interpretation of graphic components of the materials were highlighted in facilitator training. Facilitators' perspectives contributed to the review of the approach and materials. Modifications were made where necessary. A full description of the program is available [113].

## Discussion

The *WWWT *intervention meets the need for a universal non-stigmatizing approach to mental health promotion. It is based on a re-conceptualisation of the causes of postpartum depression and anxiety and therefore of the means to address the problem. The development of the intervention brings together clinical expertise, research evidence and theory from a variety of sources. *WWWT *draws on evidence about modifiable risk factors that have been overlooked in existing universal preventive strategies. It is informed by extensive contributions from the both consumers of early parenting services and heath professionals from the key relevant disciplines, clinical observations with women in early parenting services, and evidence for successful treatment interventions in these services and the community. *WWWT *is underpinned by diverse psychological and health promotion theories that provide a strong conceptual understanding of its proposed mode of action. However the model of health promotion and cultural norms on which it is based suggest that without modification the intervention may be less applicable to socioeconomically disadvantaged and minority groups than the mainstream population.

Rahman [11] has identified the key requirements for a successful mental health promotion intervention that operate at multiple levels. *WWWT *exemplifies these attributes.

### Characteristics of the *WWWT *psycho-educational intervention

Modified from Rahman (2007) [11].

Individual level

• Focus on maternal and infant health rather than maternal depression

• Modify environment rather than individual behaviour

• Involve partner and baby; be active and empowering

• Focus on relationships

• Address critical learning needs; interactive and salient

Health professional level

• Integrated into usual practice

• Enables capacity building in mental health

• Re-conceptualisation of determinants of postpartum mental health

• Challenge gender stereotypes

• Non-stigmatising

Health system level

• Evidence based

• Enhance existing health system

• Primary care, community based

• Psycho-education not mental health

• "Universal plus" stepped care pathway

• Amenable to adaptation for minority groups

*WWWT *is based on a socially-informed model of mental health operating at the individual level, which seeks to modify factors in a woman's social environment rather than within the woman herself. The intervention attends to the central features of the immediate social environment of the mother of a firstborn: her relationships with the father and with their baby. It positions postpartum mental health as a matter for families rather than individual women, and is explicitly intended to promote confident parenting and strengthen family functioning. It recognizes that first time parents' learning needs are not limited to preparation for birth, recognizes the value of fathers to maternal and infant wellbeing, and enables men to acquire specific knowledge and skills for active participation in the early life of their children. The intervention is offered in local communities and therefore affords couples the opportunity of forming social connections with other families in their area.

*WWWT *is designed to be readily integrated by health professionals into the standard program of postnatal care and offers a realistic means of mainstreaming mental health in maternal and child health services. In Victoria universal care consists of the 10 Key Ages and Stages visits and incorporates facilitated First-Time Parent Groups, which are an ideal setting for *WWWT *seminars. The compatibility of the intervention with existing programs is intended to increase the likelihood that it will be adopted by professionals. The *WWWT *facilitator training, which could readily be offered as part of professional development, offers opportunities to build mental health capacity in primary care professionals, to challenge gender stereotypes and increase awareness of the role of gender inequality as a determinant of mental health problems. It is designed to promote an appreciation of the social determinants of health and reorient practice away from screening and early intervention towards primary prevention of mental disorders in mothers of infants [5].

*WWWT *is intended to be offered as part of community based routine health care, rather than in the mental health sector, and uses a psycho-educational approach and non-psychiatric language which is likely to be less stigmatising. It could therefore enhance universal maternal and child health services and optimise the engagement of wide range of consumers, including those at heightened risk of mental health problems who are more difficult to reach. A "universal plus" model enables more vulnerable clients to be engaged in non-stigmatising standard health programs, identified early and referred for specialist mental health care. Such a "stepped" approach includes the availability of the intervention for all first time parents, and in addition, identification and referral of individual women who might benefit from additional specialist care. In the first instance *WWWT *is designed for flexible application in the dominant population, but is potentially adaptable for specific minorities including young, unpartnered, same sex partners or culturally and linguistically diverse groups, according to the needs of local communities.

## Conclusions

*WWWT *is a promising intervention for the primary prevention of mental health disorders in first time mothers of infants, which is based on evidence from multiple sources, underpinned by relevant psychological and health promotion theory, integrated into existing health services and exemplifies best practice in mental health promotion. The results of a controlled before-and-after trial of the intervention in a large socioeconomically diverse sample are reported in this journal [114].

## Competing interests

The authors declare that they have no competing interests.

## Authors' contributions

The authors contributed equally to the development of the concept, the *WWWT *intervention, and drafting the paper. Both authors read and approved the final manuscript.

## Pre-publication history

The pre-publication history for this paper can be accessed here:

http://www.biomedcentral.com/1471-2458/10/499/prepub
